# Optimizing the Clinical Impact of CAR-T Cell Therapy in B-Cell Acute Lymphoblastic Leukemia: Looking Back While Moving Forward

**DOI:** 10.3389/fimmu.2021.765097

**Published:** 2021-10-28

**Authors:** Pouya Safarzadeh Kozani, Pooria Safarzadeh Kozani, Fatemeh Rahbarizadeh

**Affiliations:** ^1^ Department of Medical Biotechnology, Faculty of Paramedicine, Guilan University of Medical Sciences, Rasht, Iran; ^2^ Student Research Committee, Medical Biotechnology Research Center, School of Nursing, Midwifery, and Paramedicine, Guilan University of Medical Sciences, Rasht, Iran; ^3^ Department of Medical Biotechnology, Faculty of Medical Sciences, Tarbiat Modares University, Tehran, Iran; ^4^ Research and Development Center of Biotechnology, Tarbiat Modares University, Tehran, Iran

**Keywords:** chimeric antigen receptor, cytokine release syndrome, neurotoxicity, acute lymphoblastic leukemia, adoptive cell therapy, cancer immunotherapy

## Abstract

Chimeric antigen receptor T-cell (CAR-T) therapy has been successful in creating extraordinary clinical outcomes in the treatment of hematologic malignancies including relapsed or refractory (R/R) B-cell acute lymphoblastic leukemia (B-ALL). With several FDA approvals, CAR-T therapy is recognized as an alternative treatment option for particular patients with certain conditions of B-ALL, diffuse large B-cell lymphoma, mantle cell lymphoma, follicular lymphoma, or multiple myeloma. However, CAR-T therapy for B-ALL can be surrounded by challenges such as various adverse events including the life-threatening cytokine release syndrome (CRS) and neurotoxicity, B-cell aplasia-associated hypogammaglobulinemia and agammaglobulinemia, and the alloreactivity of allogeneic CAR-Ts. Furthermore, recent advances such as improvements in media design, the reduction of *ex vivo* culturing duration, and other phenotype-determining factors can still create room for a more effective CAR-T therapy in R/R B-ALL. Herein, we review preclinical and clinical strategies with a focus on novel studies aiming to address the mentioned hurdles and stepping further towards a milestone in CAR-T therapy of B-ALL.

## 1 Introduction

B-cell acute lymphoblastic leukemia (B-ALL) is characterized by the presence of poorly differentiated abnormal B-cell progenitor cells that have a rapid rate of proliferation in the bone marrow (1). B-ALL is the most prevalent form of acute leukemia in children in the US (2, 3). With around 3000 newly diagnosed cases of children with B-ALL each year, overall survival (OS) surpasses 85% in children (2–4). However, in adult B-ALL patients, OS is not as favorable as it is in children with B-ALL and it ranges from 50% to up to 60% (5, 6). Even though chemotherapy regimens mediate high rates of complete remission (CR), 40% to 50% of adult B-ALL patients ultimately experience disease relapse (7–9). Relapsed or refractory (R/R) cases of B-ALL have unfavorable prognosis with CR rates ranging from around 35% and up to 10% in the first and second recovery from the disease, respectively (7–9). Therefore, researchers have been investigating other types of treatments capable of mediating better clinical outcomes and mitigated levels of side effects and adverse events.

Cancer immunotherapy first started as an idea to employ the patients’ immune system and its components as fighting tools against various types of neoplasms (10, 11). Today, monoclonal antibody (mAb)-based therapies, cancer vaccines, adoptive cell therapy (ACT), and oncolytic viruses are among famous types of cancer immunotherapy that have gained preclinical and clinical success (10). The clinical success of adoptively transferred T cells genetically engineered to express chimeric antigen receptors (CAR-Ts) has created a new era in the treatment of B-cell-based malignancies (12–17). The theory of utilizing T cells as fighting tools for a selective fight against cancer, which started in the early 1990s, has now become a treatment option for certain patients with B-cell-based malignancies (12–20). Over the past few years, the field of CAR-T therapy has progressed remarkably (11, 21). In particular, various CAR generations have been developed and many innovative strategies have been proposed, aimed at improving the safety and efficacy of CAR-T therapy (11, 20–23).

CD19-redirected CAR-T therapy of children and adults with R/R B-ALL results in high remission rates (67% to 93%) (12–17). The capacity of CD19-redirected CAR-Ts for *in vivo* activation, expansion, and robust tumoricidal activity, which results in the mentioned high rates of disease remission even in patients with R/R B-ALL, leads to adverse events and toxicities such as cytokine release syndrome (CRS), neurologic toxicities, and B-cell aplasia (14, 24–27). CRS is a systemic inflammatory condition mediated by various cytokines produced by CAR-Ts and other cells of the immune system (27, 28). C-reactive protein (CRP), interferon-γ (IFN-γ), interleukins (ILs) such as IL-1, IL-2, soluble IL-2 receptor alpha chain (IL-2Rα), IL-4, IL-6, IL-8, and IL-10, granulocyte-macrophage colony-stimulating factor (GM-CSF), granzyme B, and tumor necrosis factor-α (TNF-α) are among the most important mediators of CRS (27, 28). CRS severity can range from mild levels to high levels necessitating intensive care and urgent medical intervention (27). Moreover, CRS manifests itself as fever, tachycardia, hypotension, hypoxia, etc. (27). Additionally, the pathogenesis of CAR-T-related neurologic toxicities is not very well-known; however, it is evident that this incidence is observed in CRS-developing patients (25, 26). Neurologic toxicities can happen following or before CRS onset and it has been evident that its severity correlates with CRS severity (12, 25). Both of these adverse events are important incidences and they may limit the successful clinical outcomes of B-ALL CAR-T therapy (25, 26). It is also worth mentioning that even though most CAR-T-related adverse events are managed quite efficiently, there might still be room for improvement.

In addition to CRS and neurologic toxicities, B-cell aplasia is another CAR-T-related toxicity occurring during CAR-T therapy of B-ALL (29). B-cell aplasia is the result of on-target off-tumor toxicity of CD19-redirected CAR-Ts against normal B-cells which occurs simultaneously with the targeting of malignant CD19^+^ blasts (29). B-cell aplasia is considered a tool for measuring the persistence of CAR-Ts after obtaining the desired clinical outcomes (14, 24). Scientific evidence has demonstrated that patients with short durations of B-cell aplasia often suffer from disease relapse (24). Furthermore, B-cell aplasia puts the recipients of CD19-redirected CAR-Ts at risk of various types of infectious diseases (29); therefore, clinical interventions are highly required for mitigating the consequences of this unfavorable event.

Moreover, since the generation of autologous CAR-Ts is not always feasible, allogeneic CAR-Ts may be considered as suitable alternatives (30). However, using allogeneic CAR-Ts is also hindered by two limitations (30, 31). The first limitation is the incidence of graft-versus-host disease (GvHD) which can be life-threatening, and the second limitation is that allogeneic CAR-Ts might be rapidly attacked and eradicated by the immune system of the recipients (30, 31). Both of these hurdles significantly obstruct the tumoricidal activity of allogeneic CAR-Ts; therefore, counterstrategies are highly required for tackling these caveats (30, 31). Herein, we review recent studies that have tried to improve the clinical outcomes of CAR-T therapy in patients with R/R B-ALL by addressing the mentioned limitations. Furthermore, we also discuss preclinical and clinical studies that have investigated other factors such as media design, the duration of *ex vivo* culturing for CAR-T generation, and various phenotype-determining factors that are important for a more efficacious CAR-T therapy in B-ALL and may help achieve better clinical outcomes.

## 2 CAR-T Fundamentals

The commercial success story of CAR-Ts started in 2017 when the US Food and Drug Administration (FDA) approved the first CAR-T therapy for medical use (32). To this day, there are five FDA-approved CAR-T products available on the market for five different hematological malignancies (32–38). In particular, *tisagenlecleucel* has been approved for the treatment of certain subjects with B-ALL or diffuse large B-cell lymphoma (DLBCL) (32, 36). *Axicabtagene ciloleucel* is another CAR-T product which has been approved for DLBCL and follicular lymphoma (FL) (33, 38). In addition, *brexucabtagene autoleucel* has been approved for certain patients with mantle cell lymphoma (MCL) or B-ALL whereas *lisocabtagene maraleucel* has been FDA-approved for the treatment of certain individuals with DLBCL (37, 39). Recently, Bristol Myers Squibb’s *idecabtagene vicleucel* was FDA-approved for multiple myeloma (MM) (34, 35). *Tisagenlecleucel, axicabtagene ciloleucel, brexucabtagene autoleucel*, and *lisocabtagene maraleucel* use CD19 as their target antigen while *idecabtagene vicleucel* targets B-cell maturation antigen (BCMA) (32–38). It is important to mention that all of these products have been approved for the treatment of certain patients with particular conditions of the mentioned oncological indications (32–38).

CAR-Ts, also known as “*living drugs*”, are T cells that have been genetically manipulated to express CARs on their surface (40). These effector cells combine the precise specificity of mAbs with the cytotoxicity of T lymphocytes, without the need for major histocompatibility complex (MHC); therefore, CAR-Ts are capable of selectively targeting cells proficient in the expression of the desired target antigen while remaining unreactive towards those deficient in target antigen expression (40). CAR-Ts are either redirected towards tumor-associated antigens (TAAs) or tumor-specific antigens (TSAs) of interest expressed on the surface of tumor cells (40). In detail, cell surface-expressed TAAs or TSA are membrane-bound antigens whose expression and surface presentation is independent of MHC molecules (23). Therefore, these antigens can be targeted using mAbs and CAR-Ts (23). On the other hand, intracellular TSAs or TAAs are processed and presented by MHC molecules (41). Targeting such antigens cannot be achieved using CAR-Ts (41). Instead, T cells with engineered T-cell receptors (TCRs) specific for a particular MHC-presented peptide antigen can be beneficial (41).

CARs are synthetic receptors made of an extracellular domain (composed of a targeting domain and a hinge), a transmembrane (TM) domain, and an intracellular domain (composed of a primary T-cell activation domain and one or two co-stimulatory domains) (40). The targeting domain of CARs is mainly composed of a single-chain variable fragment (scFv) of a mAb and is responsible for the selective targeting ability of CARs (40). The hinge is responsible for attaching the targeting domain to the TM domain and is mainly derived from CD8, CD28, IgG1, or IgG4 (40). The TM domain, which acts as the bridging fragment between the extracellular domain and the intracellular domain, can be derived from molecules such as CD8α, CD3ζ, CD28, CD4, and the inducible T-cell co-stimulator (ICOS) (40). Moreover, co-stimulatory domains are considered as the helping hand of the primary T-cell activation domain responsible for CAR-T activation upon target antigen encountering (42–45). In particular, CD28, 4-1BB (CD137), OX40 (CD134), and ICOS have been used as co-stimulatory domains (42–45). It has been demonstrated that co-stimulatory domains possess properties that determine the fate of the engineered effector cells since they can induce memory or effector T-cell phenotype in CAR-Ts (1, 46, 47). Additionally, the CD3ζ part of the TCR CD3 complex, FcϵRIγ, CD3ϵ, DAP10, DAP12, the ζ-chain of TCR-associated protein kinase 70 kDa (ZAP70), the lymphocyte-specific protein tyrosine kinase (LCK), and fyn are all among the primary activation domains so far used in the construct of CAR-Ts (48–50).

**Figure 1:**
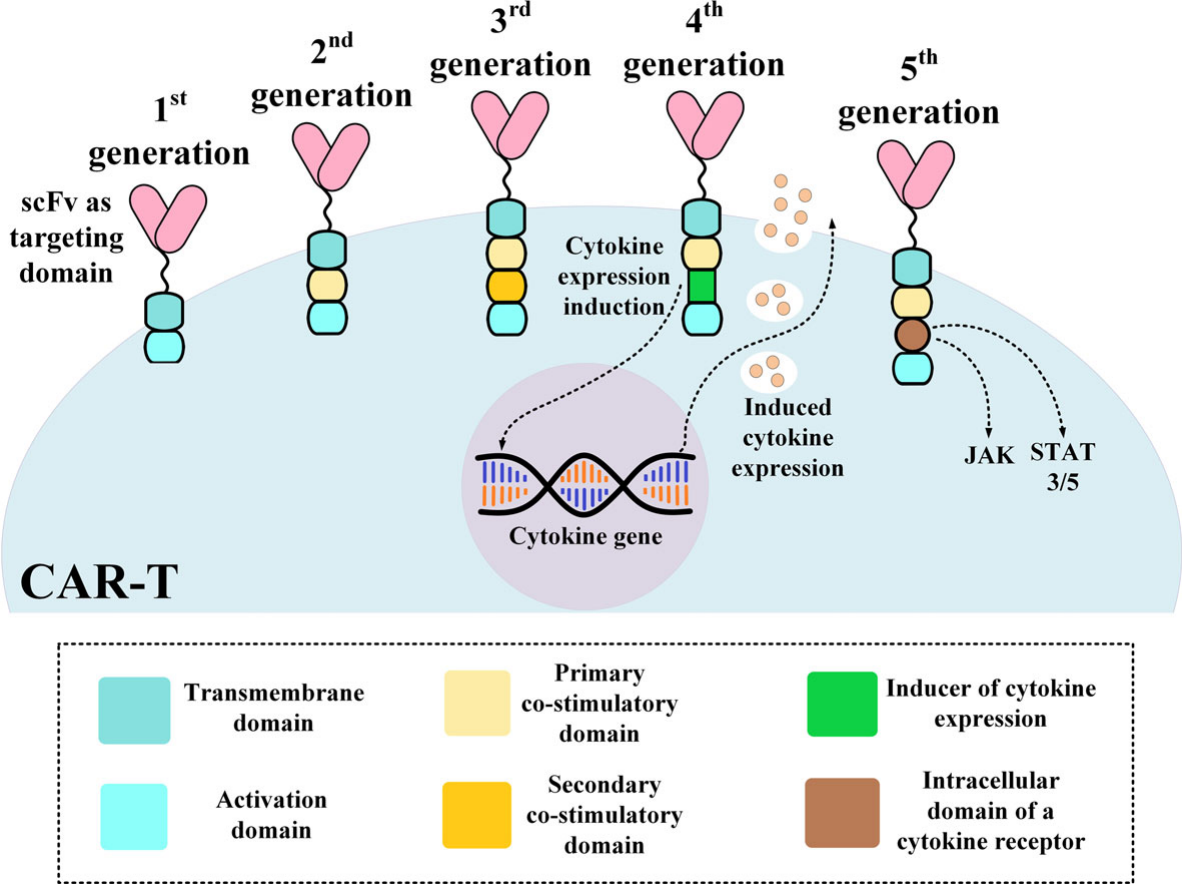
An illustration of different CAR-T generations. CAR, chimeric antigen receptor; scFv, single-chain variable fragment.

The structural evolution of CAR-Ts can be described based on their different generations. First-generation CAR-Ts had all of the mentioned domains but they did not have any co-stimulatory domain which resulted in their inadequate antitumor activity and persistence (51). As a solution, second- and third-generation CAR-Ts were designed to have one and two co-stimulatory domains, respectively, resulting in their superior antitumor activity and persistence over their predecessors (52, 53). Fourth-generation CAR-Ts are second-generation CAR-Ts that have an inducer domain for the expression of a cytokine of interest, such as IL-2, in their intracellular domain (instead of a secondary co-stimulatory domain) (54). Fourth-generation CAR-Ts are known as “*T cells redirected for universal cytokine-mediated killing*” (*TRUCKs*) or *armored CARs* since they deliver a transgenic product to the targeted tumor tissue enhancing the tumoricidal activity and efficacy of CAR-Ts (54). Fifth-generation CAR-Ts are structural counterparts of second-generation CAR-Ts but they harbor an intracellular domain of a cytokine receptor (**Figure 1**) (55, 56). With all the good news surrounding this type of therapy for B-ALL, there are remaining challenges such as CAR-T-associated toxicities including CRS and neurotoxicity, which can be mild to life-threatening, the caveats of using allogeneic CAR-Ts, and other factors whose optimization broadens the success zone of B-ALL CAR-T therapy. In the upcoming sections of this article, we will discuss strategies for addressing these hurdles.

## 3 Clinical Procedures for Improving the Efficacy and Safety of B-ALL CAR-T Therapy

### 3.1 CRS and Neurotoxicity Mitigation

CRS is the most important B-ALL CAR-T therapy-associated toxicity resulting from CAR-T-induced rapid immune activation (57–59). CRS can lead to various serious damages including cardiac toxicity, hyponatremia, and other complications (57–59). Of note, CAR-T therapy-related cardiac toxicity or cardiotoxicity is poorly recognized. However, sinus tachycardia, increase level of blood troponin, left ventricular systolic function (LVSD), profound hypotension, and decompensated heart failure (DHF) are among cardiovascular-related adverse events observed during CAR-T therapies (60). Also, hyponatremia is characterized by serum sodium (Na) levels below 135 mEq/L, and is reported to be common among patients receiving CD19-redirected CAR-T therapy (58). Moreover, neurotoxicity is another CAR-T-associated adverse event that is not yet fully known but the available data indicate that both activated CAR-Ts and endogenous T lymphocytes, and the cytokines secreted by them, may be responsible for post-CAR-T therapy neurotoxicity (61–63). Furthermore, recent single-cell RNA sequencing data have demonstrated that mural cells, that are responsible for the integrity of the brain-blood barrier (BBB), express the CD19 antigen (64). Researchers have added that the expression of CD19 in the brain emerges early in development and continues throughout adulthood (64). Additionally, these data indicate that mouse mural cells exhibit lower levels of CD19 expression which may be responsible for limitations in preclinical neurotoxicity models (64). So far, several clinical strategies, discussed in the upcoming section, have been developed to either prevent CRS and neurotoxicity or to control them and their damaging effects. Furthermore, designing approaches can also be beneficial in the prevention of CAR-T-associated toxicities (50). For instance, instead of the generally used CD3ζ activation domain, researchers have designed and generated CAR-Ts with DAP12 as the activation domain (50). In detail, these second-generation CAR-Ts are made of the natural killer group 2D (NKG2D) ectodomain fused to 4-1BB and the DAP12 cytoplasmic domain (which acts as the activation domain) (50). This design resulted in reduced levels of IFN-γ, TNF-α, and IL-2 release during tumor cell lysis with lower proliferative activity upon repeated antigen stimulation but similar tumoricidal activity in comparison with that of NKG2D CAR-Ts with the CD3ζ activation domain (50). Naturally, NKG2D receptor has the role of a primary activation signal in natural killer (NK) cells whereas it acts as a co-stimulatory signal in T lymphocytes (65). In detail, the NKG2D receptor is a homodimer molecule that interacts with the adaptor molecules DAP10 or DAP12 based on the expressed NKG2D isoform (65). Moreover, mouse and human NK cells, T cells (αβ and γδ), and natural killer T (NKT) cells express a longer isoform of NKG2D that interacts with DAP10 (65). In addition, mouse NK cells also express a shorter splice variant of NKG2D that associate with DAP12 (65). Both DAP10 and DAP12 are responsible for triggering downstream cell signaling cascades in association with NKG2D upon ligand encountering (65).

#### 3.1.1 IL-1 and IL-6 Blockade

Recently, studies have shown that IL-1 and IL-6 released by monocytes and macrophages are the main reasons for CAR-T-associated CRS and *immune effector cell-associated neurotoxicity syndrome* (*ICANS*) (66, 67). In detail, monocytes play a more important role than macrophages in CRS-progressing IL-1 and IL-6 secretion (67). Preclinical data indicate that monocyte ablation or IL-6 receptor blockade using *tocilizumab* can prevent CRS but not lethal neurotoxicity (67). This is because IL-6 receptor blocking antibodies broadly used as the standard procedure for the clinical management of severe CRS cannot penetrate the BBB which results in their inability to induce complete *CAR-T-related encephalopathy syndrome* (*CRES*) remission (67). Furthermore, CRS and CRES are the major safety-related issues of CAR-T therapy which are caused by a rapid increase in the level of multiple cytokines, mainly IL-6, secreted by the activated infused CAR-Ts and mononuclear cells including dendritic cells and macrophages (68). In detail, target cell-induced IL-6 expression and production by CAR-Ts trigger factors that enhance monocyte production and release of IL-6 and other proinflammatory cytokines contributing to CRS and CRES progression (69). Therefore, some researchers have investigated a very novel strategy to address these limitations (69–71). It has been discovered that suppressing the IL-6 gene expression in CAR-Ts significantly decreases the IL-6 release from monocytes and it also reduces the possibility of severe CRS and CRES without reducing the antitumor efficacy of CAR-Ts *in vitro* and *in vivo* (69, 70). Therefore, one research group has generated a CAR construct with IL-6 shRNA (70). The shRNA-IL6-modified CD19-redirected CAR-Ts (ssCAR-T-19) were delivered to a B-ALL patient with skin and testicle extramedullary relapse enrolled in a clinical trial (NCT03919240) (70). The patient was successfully treated by ssCAR-T-19s and this study proposed that these CAR-Ts might be therapeutically useful in efficiently eradicating infiltrating leukemia cells in the skin and testicle with a mild level of toxicity (however, broader patient populations and more careful clinical investigations are required for drawing substantiated conclusions) (70). Furthermore, ssCAR-T-19s have also been investigated in a recent clinical trial (NCT03064269) involving three patients with relapsed central nervous system (CNS) B-ALL (71). The results of this study demonstrated that ssCAR-T-19s can migrate into the CNS and reduce brain leukemic infiltration and significantly eradicate leukemic blasts in the cerebrospinal fluid (CSF) with mildly elevated cytokine levels and only grade 1 CRS (71). Therefore, this method could be proposed as an approach for reducing the risk of CRES in CAR-T therapy of patients with CNS B-ALL (71). However, in the case of severe CRS occurrence, IL-6 receptor blockade or IL-6 expression inhibition strategies are known to be inadequate approaches (16, 72). Therefore, it is necessary to use high-dose corticosteroid drugs to efficiently control the progression of life-threatening CRS (16, 72). Moreover, recent studies have shown that corticosteroids do not influence the efficacy and kinetics of CAR-Ts in the treatment of B-ALL (73). Additionally, *TO-207* is an investigational drug that can inhibit the abnormal activation of macrophages and monocytes with no negative effects on the activity of T cells (74–77). *In vitro* studies have demonstrated that *TO-207* acts as a multi-cytokine inhibitor by suppressing monocyte-mediated secretion of IL-6 and other inflammatory cytokines (including IL-1β, monocyte chemoattractant protein 1 (MCP-1), IL-8, IL-18, and GM-CSF) without deteriorating CAR-T functionality (74, 78). Furthermore, IL-1 receptor blockade using the IL-1 receptor antagonist immunosuppressive drug *anakinra* has been an effective approach resulting in encouraging preclinical outcomes by preventing both lethal neurotoxicity and CRS (67). The same study has also claimed that *anakinra* and *tocilizumab* exhibit the same level of effectiveness in IL-6 signaling blockade and lethal CRS prevention in preclinical models (67). Moreover, CAR-Ts particularly engineered to secrete IL-1 receptor antagonists have also been investigated in preclinical studies (66). The results have indicated that IL-1 receptor antagonist-secreting CAR-Ts can prevent or mitigate CRS as well as neurotoxicity in a very efficient manner (66). As discussed, IL-1 blockade strategies can be efficiently utilized to prevent life-threatening CAR-T-related toxicities including CRS and neurotoxicity (66). However, it is important to mention that conducting clinical investigations with a high number of patients is a vital step in further validating the preclinical and clinical outcomes discussed in this section.

#### 3.1.2 GM-CSF Blockade

Neurotoxicity and CRS development after CAR-T therapy are directly related to *in vivo* T-cell expansion and subsequent secretion of T-cell effector cytokines such as IL-6, IFN-γ, GM-CSF, and MCP-1 suggesting that even monocytes and macrophages may have contributions in the emergence of such adverse events (15, 79–82). Therefore, the neutralization of such cytokines may be a potential strategy for the management of these CAR-T-related toxicities (79, 83, 84). GM-CSF neutralization reduces the secretion level of CRS-mediating cytokines such as IL-6, IL-8, and MCP-1 (79, 83, 84). Therefore, reductions in the level of these immune cell trafficking mediators can decrease the number of CNS-infiltrating immune cells which results in a reduced level of neuroinflammation (NI) and CRS (79, 83, 84). Sterner et al. have evaluated the effects of GM-CSF neutralization on the functionality of CD19-redirected CAR-Ts using *lenzilumab* and they have demonstrated that *lenzilumab* does not inhibit the function of the CAR-Ts (79). Moreover, they have reported enhanced CD19-redirected CAR-T proliferation and durable control of leukemic disease in patient-derived xenograft (PDX) models after *lenzilumab*-mediated neutralization of GM-CSF (79). Additionally, these researchers expanded their experimental zone by generating CRISPR-Cas9-mediated GM-CSF secretion-deficient (GM-CSF^k/o^) CD19-redirected CAR-Ts (79). These GM-CSF^k/o^ CAR-Ts were capable of maintaining their normal functions, exhibiting enhanced *in vivo* tumoricidal activity, and improving OS as compared with conventional CD19-redirected CAR-Ts (79). Other studies have used other genome-editing strategies such as the *transcription activator-like effector nuclease* (*TALEN*) for the same aim (85). They have demonstrated that their GM-CSF^k/o^ CAR-Ts can mediate a substantially reduced secretion level of GM-CSF leading to a disrupted or decrease macrophage-dependent secretion of CRS-mediating factors such as MCP-1, IL-6, and IL-8 (85). Moreover, CAR-Ts can be engineered to be deficient in GM-CSF expression and produce anti-GM-CSF mAbs. The mentioned findings demonstrate that GM-CSF inhibition might be considered as a potential option for the abrogation of CRS and NI and enhancement of CAR-T function (79). However, it is safe to conclude that in-depth clinical assessments might help researchers understand the suitability of such strategies in clinical settings.

#### 3.1.3 Catecholamine Blockade

Myeloid-derived catecholamines are known as crucial mediators of CRS (86–88). Macrophages secrete and respond to catecholamines *via* their adrenergic receptors upon encountering inflammatory stimuli (86–88). This mechanism results in an increase in the level of cytokine production and secretion (87–89). Therefore, high levels of circulating catecholamines can lead to excessive inflammatory responses (87–89). It has been found that disrupting the key enzymes involved in the synthesis of catecholamines can reduce its circulating level and prevent CRS occurrence (90, 91). For example, myeloid-specific deletion of tyrosine hydroxylase (TH) using *metyrosine* (*MTR*) can reduce the excessive level of this key CRS mediator without impairing CAR-T antitumor activity (91). Additionally, *atrial natriuretic peptide* (*ANP*) can also significantly reduce the circulating level of catecholamines without any interference with CAR-T-mediated therapeutic responses (90).

#### 3.1.4 Application of JAK Inhibitors

As key CRS modulators, inflammatory cytokines including ILs, IFNs, and several growth factors crucially require the JAK tyrosine kinase family for their downstream signaling pathway (92–99). Therefore, the inhibition of the JAK family components can lead to a reduced CRS-related cytokine level (92–99). *Itacitinib* (INCB039110) is a JAK1 inhibitor that is being evaluated in clinical trials for the treatment of GvHD (NCT02614612) (99) and in preclinical investigations for the treatment of inflammatory and autoimmune diseases (97, 98). *Itacitinib* has also been evaluated in combination with other drugs for the treatment of various B-cell malignancies in clinical trials (NCT02018861 and NCT01905813) (95, 96). Recently, studies have evaluated the effects of prophylactic *itacitinib* in the prevention of CAR-T therapy-mediated CRS, and they have found that *itacitinib* does not impair CAR-T functionality both *in vitro* and *in vivo* and it significantly reduces CRS-associated cytokine levels in a dose-dependent fashion (94). Such data suggest the potential of *itacitinib* as prophylactic therapy for the prevention of CAR-T-related CRS as other studies investigated its various safety aspects (93). Additionally, *ruxolitinib* is also another JAK inhibitor that has recently been used as adjuvant therapy in a case report study involving a patient with B-ALL (92). *Ruxolitinib* has been successful in controlling corticosteroid-resistant CRS without interfering with CAR-T functionality (92). Such studies highlight the role of JAK inhibitors in the prevention and control of CRS and pave the way for more in-depth preclinical and clinical investigations.

#### 3.1.5 Intrathecal (IT) Chemotherapy

IT chemotherapy has been used for controlling refractory CAR-T-associated ICANS that is non-responsive to steroids (100, 101). Shah et al. have administered IT chemotherapy to two CAR-T-receiving patients and demonstrated that it can mediate rapid ICANS resolution without any long-term complications (100). This method can also accelerate the recovery process and reduce the complications of systemic long-term corticosteroid administration (100). Moreover, Yucebay et al. have also reported similar results from a study where IT chemotherapy resulted in the resolution of high-grade neurotoxicity (grade 3-4) of CD19-redirected CAR-T-receiving patients while corticosteroid therapy was inefficient (101). However, both of these studies suggest that this method requires detailed evaluations in future clinical trials (100, 101).

#### 3.1.6 Pretreatment With Antibody-Based Immunotherapy

The occurrence of CRS and neurotoxicity seem to have a direct relationship with the disease burden of patients receiving CAR-T therapy (14, 16, 17). Pretreating patients with antibody-based immunotherapy such as *blinatumomab* or *inotuzumab ozogamycin* (*IO*) before the administration of CAR-Ts has been known to potentially reduce disease bulk, thus minimizing the incidence and severity of the mentioned toxicities (102). *Blinatumomab* is a T-cell-redirecting bispecific antibody (TRBA) that can potentially mediate the elimination of leukemic cells through the engagement of endogenous T cells by simultaneously binding to CD19 on the surface of B-lineage cells and CD3 on cytotoxic T cells (103). Moreover, *IO* is an antibody-drug conjugate consisting of a humanized mAb against CD22 conjugated to the cytotoxic antibiotic agent *ozogamycin* (104). These therapeutic antibodies have shown considerably improved OS rates in comparison with standard therapy (103, 104). Additionally, one study including five adult patients with B-ALL (NCT02772198) has reported that treatment of R/R B-ALL patients with *blinatumomab* and/or *IO* before the beginning of CD19-redirected CAR-T therapy can result in promising response rates (with two out of five patients (40%) achieving minimal residual disease (MRD)-negative CR and two other patients (40%) achieving MRD-positive CR) (102). However, further investigations involving broader patient populations are required for a better understanding of the action mechanisms of antibody-based treatments before CAR-T therapy and their effects on the final clinical outcomes.

#### 3.1.7 Therapeutic Plasma Exchange (TPE)

**Figure 2:**
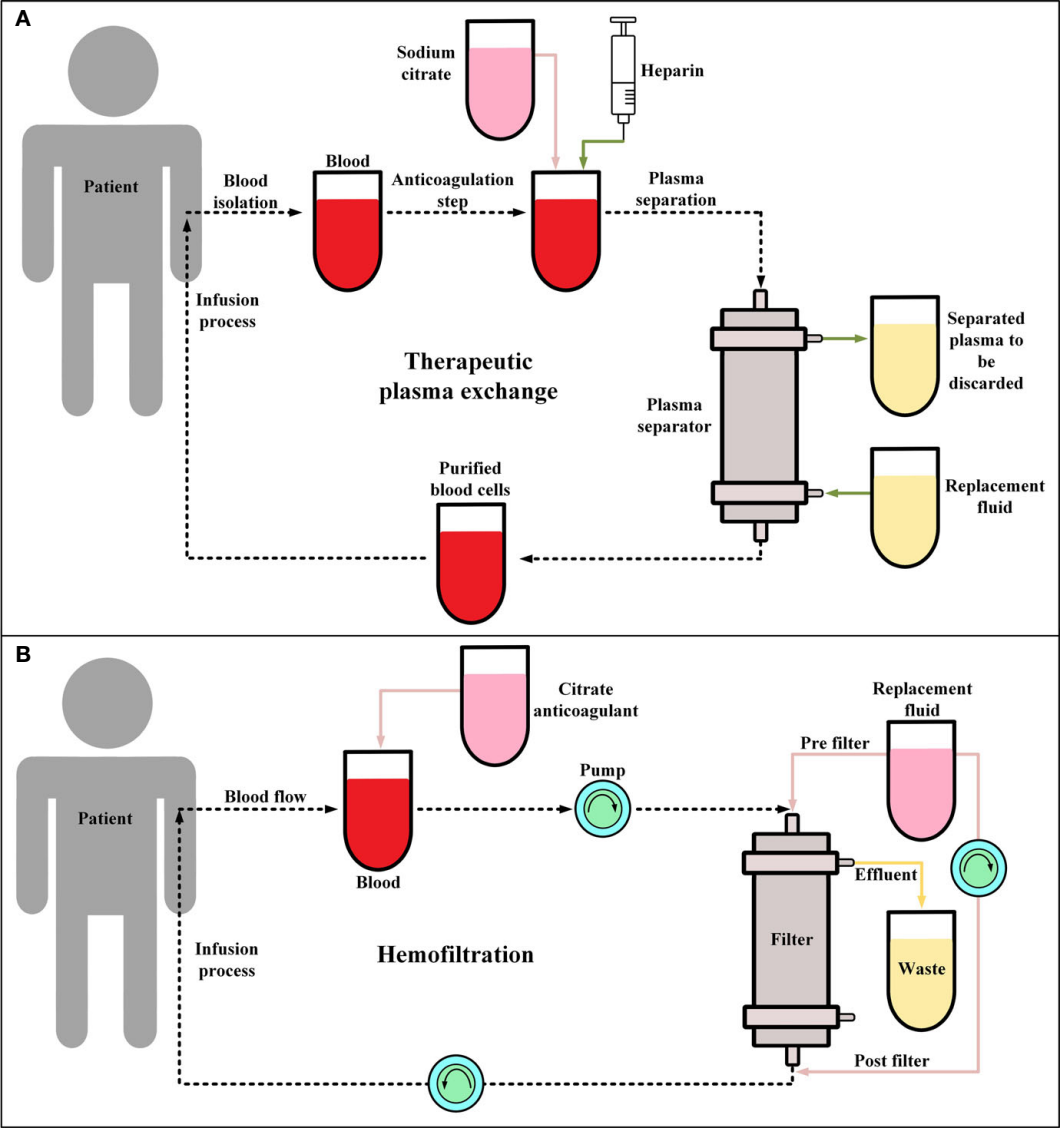
*Therapeutic plasma exchange* (TPE) and *hemofiltration*. **(A)** The general procedure of TPE. Conventionally, in the anticoagulation step, sodium citrate or heparin are utilized. Moreover, the replacement fluid consists of albumin or fresh frozen plasma. **(B)** The procedure of *continuous veno-venous hemofiltration* (CVVH) for hemofiltration. During CVVH, a patient’s blood is filtered by the means of highly semipermeable membranes (known as filter) and the ultrafiltrate (wastes) is separated by a process known as convection. Of note, the replacement fluid is added prior to or after hemofiltration.

Recently, it has been shown that TPE (**Figure 2A**) in combination with glucocorticoid therapy can result in the gradual resolution of the CRS-related symptoms of CAR-T therapy recipients (105, 106). These findings suggest that this strategy can be a feasible procedure, at least in patients with severe CRS (grade ≥ 3), even though TPE is not included in CRS management guidelines (105, 106). In a case report, Xiao et al. reported that in a 23-year-old male R/R B-ALL patient, after performing TPE along with the administration of *dexamethasone*, CRS (grade 3) was mitigated and controlled whereas treatment with antiallergic and antipyretic drugs, glucocorticoids, and *tocilizumab* was not effective against the progressing condition of the patient’s CRS (105). Moreover, another case report has also reported that TPE has been effective in the treatment of CAR-T-induced CRS of a 4-year-old female patient with R/R B-ALL (107). Additionally, the report of a clinical trial (NCT02349698) involving ten patients has indicated that *tocilizumab*, glucocorticoid, and TPE have been effective in the successful controlling of severe CRS (grade 3-4) developed in four of the patients (108). However, the broader application of this approach for the management of CAR-T-related CRS lies in the outcomes of more comprehensive future clinical studies in this regard.

#### 3.1.8 Hemofiltration

Hemofiltration (**Figure 2B**) has not been generally utilized as a clinical approach for controlling CAR-T therapy-related CRS. However, studies have used hemofiltration to help patients with high-grade CRS (grade ≥4), with developed acute kidney injury, to recover their renal functionality (109). Moreover, a case report has indicated that hemofiltration has been helpful in mitigating severe CRS (grade 4) and preventing multiple organ failure and pulmonary infection in a B-ALL patient after CD19-redirected CAR-T therapy (110). It is important to state that in this case, *tocilizumab* and glucocorticoids were not successful in controlling the mentioned adverse events (110). Furthermore, another study has also proposed that *continuous renal replacement therapy* (*CRRT*) can be an additional approach for controlling CAR-T therapy-related CRS that is resistant to conventional treatment (111). This study has demonstrated that *CRRT* is effective in mitigating sepsis which has a similar pathophysiological mechanism to CRS (111). However, as such studies themselves indicate that there are still various unanswered questions in this field that require addressing (111).

#### 3.1.9 Fractionated CAR-T Infusion

Recently, studies have indicated that there is a relationship between CAR-T infusion dose and the occurrence of CRS (112–114). Therefore, a fractionated dosing scheme can retain high response rates with acceptable tolerability in adult R/R B-ALL patients (113, 114). So far, two clinical trials have evaluated this strategy. The first study (NCT01029366) included R/R B-ALL patients alongside patients with other CD19-positive malignancies and the second one (NCT02030847) only included adults with CD19^+^ R/R B-ALL (113, 114). In detail, Frey et al. administered *tisagenlecleucel* to adult R/R B-ALL patients after lymphodepletion as either a one-time infusion or fractionated infusions split over 3 days (10% on day 1, 30% on day 2, and 60% on day 3) (113). The total planned CAR-T infusion dose in their study varied with adaptive protocol modifications in response to efficacy and CRS occurrence (113). The second clinical trial included 35 patients in 3 different dosing cohorts which included the low-dose cohort (9 patients), the high-dose single infusion cohort (6 patients), and the high-dose fractionated (HDF) cohort (20 patients) (113). In the low-dose cohort, the patients received single or fractionated dosing, which resulted in manageable toxicity with a 33% CR rate (113). In the high-dose single infusion cohort, half of the patients died due to refractory CRS (grade 4/5) and culture-positive sepsis, and the other half achieved CR (113). Moreover, the HDF cohort had a 90% CR rate and manageable CRS (grade ≤3) (113). Of note, according to Penn Grading System for CRS, grade 4 is characterized by life-threatening complications including hypoxia which necessitates the appliance of mechanical ventilation and hypotension requiring the administration of high-dose antihypotensive agents. Also, grade 5 is defined as CRS-caused mortality (113). The HDF scheme resulted in the highest survival rate with a 2-year OS of 73% and event-free survival of 49.5% (113). The researchers of this study proposed that a fractionated dosing scheme of CTL019, for administration to adult R/R ALL patients, with intrapatient dose modifications can optimize the safety of B-ALL CAR-T therapy without impairing its efficacy (113). Additionally, these clinical trials have demonstrated that a fractionated dosing scheme, where day 2 and day 3 doses are held in response to early CRS, enables individualized dose modifications for achieving a better balance of efficacy and safety in comparison with protocols suggesting a defined single-dose infusion (113).

### 3.2 Overcoming Graft Rejection

Using autologous CAR-Ts in clinical settings may not always be feasible since the patients may be under different types of treatments and may have malignancies with various levels of severity which might imping on the quality of the final T-cell products and the corresponding clinical outcomes (115). Considering these limitations, allogeneic CAR-Ts, generated from donor-derived T cells, can be used as an alternative even though they tend to have their limitations (115). Such limitations include causing alloreactivity-related adverse events such as host-versus-graft (HvG) and graft-versus-host (GvH) reactions (115). In this section, we will review studies focusing on developing strategies for addressing these limitations.

#### 3.2.1 Conditioning Regimen

Conditioning regimen, also known as lymphodepleting conditioning regimen, is the occasional use of chemotherapeutic agents in cell-based cancer therapies such as CAR-T therapies (regardless of autologous or allogeneic CAR-T therapies) (116). It has been demonstrated that conditioning regimen is an important factor affecting the clinical outcomes of CAR-T therapy (116). Studies have demonstrated that conditioning regimen prior to adoptive cell transfer significantly enhances the efficacy of therapies with *ex vivo*-expanded tumor-infiltrating lymphocytes (TILs) (117). In brief, lymphodepletion chemotherapy provides an appropriate environment for infused T cells to expand by helping create a supportive space in the immune system of the recipients (116–119). Lymphodepleting conditioning regimen acts by several mechanisms including eliminating the recipients’ T and B lymphocytes as well as NK cells, the eradication of immunosuppressive cells such as regulatory T cells and myeloid-derived suppressor cells, inducing co-stimulatory molecules and downregulating indoleamine 2,3-dioxygenase (IDO) in tumor cells, the elimination of homeostatic cytokine sinks such as IL-2, IL-7, and IL-15, and finally promoting the expansion, function, and persistence of the adoptively transferred T cells by eliminating the factors interfering with their activity (117–119).

*Fludarabine* and *cyclophosphamide* are two chemotherapeutic agents that are occasionally used as the conditioning regimen for B-ALL patients receiving CAR-T therapy (120, 121). Studies have demonstrated that using the combination of these drugs in the lymphodepleting conditioning regimen would result in improved CD19-redirected CAR-T expansion and persistence, better clinical outcomes, and disease-free survival of the patients in comparison with non-*fludarabine* lymphodepleting regimens (120, 121). Others have shown a relationship between elevated in-serum IL-15 levels and clinical response after CD19-redirected CAR-T therapy (122). It is also believed that the probability of achieving a favorable cytokine profile is higher in patients who receive high-intensity lymphodepletion in comparison with those that receive low-intensity lymphodepletion (123). This occurrence in turn is associated with better clinical outcomes (123).

#### 3.2.2 Using Genome-Editing Methods

Genome-editing technologies have been utilized for addressing the unfavorable limitations of using allogeneic T cells (124–127). In this regard, Torikai et al. have utilized the zinc finger nuclease (ZFN) genome-editing technique to eliminate the expression of the endogenous αβ TCR in allogeneic CD19-redirected CAR-Ts (128). They demonstrated this method can prevent allogeneic CAR-T-mediated GvHD without compromising CAR-T functionality or antitumor activity (128). Stepping further, they used the same genome-editing method and disrupted the expression of HLA-A in CD19-redirected CAR-Ts, and demonstrated that this strategy can protect CAR-Ts from lymphodepletion therapy, alongside eliminating the possibility of GvHD (129).

Other researchers have developed practical platforms to demonstrate the applicability of the TALEN genome-editing technique for the production of “off-the-shelf” allogeneic CAR-Ts (130–132). In this regard, Poirot et al. used TALEN for the simultaneous disruption of two genes in allogeneic CAR-Ts (130). First, the researchers disrupted the TCRα constant (TRAC) gene expression which resulted in the elimination of TCRαβ expression; therefore, it abrogated the potential of the allogeneic CAR-Ts for GvHD mediation (130). The second knock-out was aimed at rendering the universal allogeneic CAR-Ts resistant to destruction by a lymphodepleting or immunosuppressive agent (130). CD52, as the second knock-out target, can be specifically targeted using the lymphodepleting mAb *alemtuzumab*, thus enabling the lymphodepletion of recipient(s) and the creation of a receptive environment concurrently or before the administration of the TCR/CD52-knock-out CD19-redirected CAR-Ts (130). Poirot et al. demonstrated that the TCR/CD52-knock-out CD19-redirected CAR-Ts were capable of efficient elimination of CD19^+^ tumor targets even while accompanied by the chemotherapeutic agent (130). Taken together, this GMP-compatible scalable process can be considered as a general platform for the manufacturing of “off-the-shelf” CAR-Ts from the T cells of healthy third-party donors (130). Similarly, Qasim et al. also generated allogeneic TCR/CD52-knock-out CD19-redirected CAR-Ts from non-HLA matched donors, and demonstrated that these genome-edited universal allogeneic CAR-Ts did not mediate alloreactivity in infant recipients with R/R B-ALL (132). They also reported that these CAR-Ts mediated molecular remission in the recipients with acceptable persistence while being anti-CD52 therapy-resistant (132). Moreover, other researchers have also demonstrated that simultaneous knock-out of TCRαβ expression and deoxycytidine kinase (dCK) can result in the production of allogeneic CD19-redirected CAR-Ts that do not mediate GvHD and are resistant to dCK phosphorylation-dependent lymphodepleting regimens (131).

It is worth mentioning that the results of two Phase I trials using TCR-knockout CD19-redirected CAR-T therapy in both children and adults (NCT02808442 and NCT02746952, respectively) have demonstrated the practicability of using allogeneic genome-edited CAR-Ts for the treatment of patients with R/R B-ALL (133). The results of such trials indicate that allogeneic CAR-Ts can mediate acceptable tumoricidal activity with manageable levels of toxicities and adverse events (133). Such CAR-Ts can be beneficial for the treatment of heavily pretreated R/R B-ALL patients with rapidly progressing diseases since autologous CAR-Ts may not be accessible in these cases in terms of meeting the adequate cell number or final-product quality (133).

In addition to the abovementioned strategies, CRISPR-Cas9-based genome-editing method can also be utilized for the generation of universal allogeneic CAR-Ts (134–137). One study has used CRISPR-Cas9 to selectively insert the CD19-redirected CAR transgene into the TRAC locus (134). This mechanism results in the deficiency of the endogenous TCR expression and it ameliorates *in vivo* CAR-T functionality while reducing the risk of alloreactivity as compared with retrovirus-mediated random CAR transgene insertion (134). It also postpones the effector T-cell differentiation and exhaustion of CAR-Ts and facilitates the efficient internalization and consequent re-expression of CAR molecules upon multiple target antigen engagement (134). MacLeod et al. made a similar attempt and demonstrated that their TCR-knock-out CD19-redirected CAR-Ts exhibited superior antitumor activity in preclinical mouse models without mediating GvHD (136). Furthermore, researchers incorporated multiple gRNAs into a CAR-encoding lentiviral vector which resulted in the simultaneous knock-out of the endogenous TCR and Beta-2 microglobulin (b2M) of HLA class I, thereby paving the way for a practical strategy for the production of universal allogeneic CAR-Ts (137).

More recently, Georgiadis et al. demonstrated that incorporation of an sgRNA element into the Delta U3 3′ long terminal repeat (LTR) of their CAR-encoding lentiviral vector would result in the production of a self-inactivating lentiviral terminal vector that combines CAR expression with CRISPR-Cas9 effects (135). In detail, after transcription and duplication of the hybrid DU3-sgRNA and electroporation-facilitated delivery of Cas9 mRNA, this platform cleaves the TRAC locus and enables enriching the TCR-knock-out CAR-T population using automated magnetic separation (135). These CAR-Ts would retain their CAR-associated specificity and antileukemic activity, have superior persistence as compared with their conventional counterparts, and would not mediate alloreactivity in the respective recipients (135). However, despite what we have discussed, a recent study by Stenger et al. has indicated that CD19-redirected CAR-Ts harboring endogenous TCR have superior persistence and mediate considerably prolonged leukemia control *in vivo* as compared to their CRISPR-Cas9-mediated TCR-knockout counterparts (138). Conclusively, it can be mentioned that more clinical information is required for drawing more substantiated conclusions.

#### 3.2.3 Virus-Specific T Lymphocytes (VSTs) for CAR Expression

VSTs that harbor their native TCRs can be used as a source of universal allogeneic T cells in CAR-T therapy (139, 140). *Tabelecleucel* (tab-cel^®^) is a universal allogeneic T-cell immunotherapy using Epstein-Barr virus (EBV)-specific T cells that harbor TCRs specifically targeting EBV antigens (139, 140). It has been demonstrated that CD19-redirected CAR-Ts generated using EVB-specific T cells are well-tolerated with poor mediation of GvHD and CRS suggesting a promising approach for generating universal allogeneic CAR-Ts (140).

VSTs are now being investigated for the prevention of viral infections in recipients of hematopoietic stem cell transplantation (HSCT) since these cells can reconstruct the recipients’ impaired immune system and confer immunity against life-threatening viral infections after HSCT without GvHD mediation (141, 142). This feature of VSTs has encouraged researchers to use them as a reliable source for the generation of allogeneic CAR-Ts (142, 143). An ongoing Phase I clinical trial (NCT00840853) has studied VSTs genetically engineered to express CD19-redirected CARs to see if these cells can maintain their antiviral activity alongside exhibiting specific tumoricidal functionality in patients with CD19^+^ B-ALL after HSCT (142). According to the results of this clinical trial so far, the investigators stated that these CAR-Ts induced no infusion-related toxicities (142). The researchers have also added that vaccinating the recipients of these CAR-Ts with viral antigens may result in enhanced CAR-T expansion and antitumor activity (142). However, this clinical trial is planned to be completed in 2031; therefore, more accurate clinical outcomes can be obtained by then.

#### 3.2.4 Human Induced Pluripotent Stem Cell (hiPSC)-Derived CAR-Ts

Lately, hiPSC-derived CAR-Ts have gained a great deal of attention as universal allogeneic CAR-Ts (144, 145). In detail, peripheral blood-derived T cells are converted to hiPSCs and genetically engineered to express the CD19-redirected CAR with targeted integration into the TRAC locus followed by clonal selection carried out for generating a master hiPSC line, termed TRAC-TiPSC (144, 145). These cells are also genetically manipulated to express a high-affinity form of CD16 with proteolytic resistance for addressing tumor antigen escape (144, 145). The mentioned characteristics of hiPSC-derived CAR-Ts render them incapable of GvHD mediation (144, 145). Taken together, the researchers of these studies indicated that since such CAR-Ts mediated encouraging preclinical results as an effective and safe allogeneic product, it might be feasible to evaluate these CAR-Ts in a Phase I clinical trial in patients with B-cell neoplasms (144, 145).

#### 3.2.5 Placenta Blood-Derived T Cells for CAR Expression

Recently, researchers have demonstrated that T cells isolated from postpartum human placenta/umbilical cord blood can be used for the generation of CD19-redirected CAR-Ts, termed P-CD19-redirected CAR-Ts (146). P-CD19-redirected CAR-Ts are mainly naïve (CD45RA^+^) T cells and retain their naïve/memory marker expression and harbor a lower expression of effector/exhaustion markers as compared to peripheral blood mononuclear cell-derived CAR-Ts (146). Moreover, these cells also have superior immune tolerance to HLA mismatch and exhibit weakened allogeneic activation resulting in a lower GvHD occurrence possibility (146). Researchers have suggested that these features of P-CD19-redirected CAR-Ts make them a promising candidate for universal allogeneic CAR-T therapy (146). It is worth mentioning that the endogenous TCR expression of these T cells can be abrogated using the CRISPR genome-editing technique to minimize the risk of endogenous TCR-mediated GvHD (146).

#### 3.2.6 Alloimmune Defense Receptor (ADR)

One strategy for overcoming the issue of cellular immune rejection is the abrogation of immune responses mediated by both activated alloreactive T cells and NK cells that direct the elimination of foreign cells through different mechanisms (147–151). Mo et al. have developed an engineered receptor called ADR that mediates the depletion of activated host T and NK cells through selective recognition of the cell surface receptor 4-1BB since this molecule is temporarily upregulated on the cell surface of activated CD4^+^ T cells and CD8^+^ T cells, as well as NK cells (151). The 4-1BB-specific chimeric ADR is composed of a 4-1BB-recognizing fragment derived from 4-1BB ligand (4-1BBL) that is connected to the intracellular CD3ζ chain through a spacer and a TM region (151). *In vitro* and *in vivo* results have indicated that ADR-expressing T cells manage to resist cellular rejection by targeting alloreactive lymphocytes and, on the other hand, they manage to spare resting lymphocytes (151). Furthermore, Mo et al. co-expressed ADR alongside second-generation CD19-redirected CARs, and demonstrated that T cells co-expressing these two chimeric receptors can preserve their independent anti-rejection and antitumor functionality (151). Moreover, T cells expressing only CD19-redirected CARs and T cells co-expressing both CD19-redirected CARs and ADRs have exhibited similar cytotoxic profiles against CD19^+^ cells, according to Mo and colleagues (151). Conclusively, these researchers have proposed that co-expression of CARs and ADRs can mediate sustained tumor eradication and produce long-term therapeutic benefit in immunocompetent recipients with hematologic malignancies alongside enabling the generation of rejection-resistant off-the-shelf allogeneic CAR-Ts (151).

#### 3.2.7 HLA-Matched or HLA-Haploidentical CAR-Ts

Using HLA-matched or HLA-haploidentical allogeneic CAR-Ts can lead to different clinical outcomes in B-ALL CAR-T therapy (106). Researchers have investigated the use of HLA-matched or HLA-haploidentical allogeneic CD19-redirected CAR-Ts (M-CAR-Ts and H-CAR-Ts, respectively) for the treatment of R/R B-ALL patients to evaluate which one can result in better clinical outcomes (106). Recently, Jin et al. described the first-in-human application of M-CAR-Ts in R/R B-ALL patients before allogeneic hematopoietic stem cell transplantation (allo-HSCT) (106). The results of this investigation demonstrated that using M-CAR-Ts for the treatment of R/R B-ALL patients results in higher CR rates but it also leads to more severe toxicities in comparison with using H-CAR-Ts (106). These researchers also reported that the mild infusion-related toxicities occurring in patients receiving H-CAR-Ts enabled using higher dose infusions in these patients in comparison with the patients receiving M-CAR-Ts (106). It is important to mention that even though these researchers did not observe GvHD or any uncontrolled infusion-related toxicities, there is still a need for paying considerable attention to GvHD and other infusion-related toxicities (106). Other researchers have also investigated the use of H-CAR-Ts (152–154). In particular, Cai et al. and Zhang et al. have reported that using H-CAR-Ts has beneficial therapeutic effects as part of a conditioning regimen for allo-HSCT (152, 153). However, it is suggested that, as compared to M-CAR-Ts, the lower efficacy of H-CAR-Ts may be due to their heterogeneity in mediating graft rejection (106). Additionally, it is yet to be discovered that whether using a stronger immunosuppressive treatment before the infusion of H-CAR-Ts can enhance the efficacy and antitumor activity of these cells (106).

### 3.3 Overcoming B-Cell Aplasia and Its Consequences

B-cell aplasia, as characterized by the low number or the absence of B cells, is commonly considered as an indicator of a successful CD19-redirected CAR-T therapy which can last for as long as CAR-Ts are potentially functional (29). B-cell aplasia can lead to agammaglobulinemia and hypogammaglobulinemia resulting in an increased risk of sinopulmonary and various other life-threatening infections due to the inability of the patients’ immune system to produce antibodies (29). Prevention of serious bacterial infections in patients with hypogammaglobulinemia after CD19-redirected CAR-T therapy and also in patients with primary immunodeficiency (PID) who suffer from impaired immune systems can be achieved through immunoglobulin replacement (14, 15, 155–158). Increasing the level of serum immunoglobulin G (IgG) has been correlated with a considerably lower risk of sinopulmonary infection (29). Serum IgG level of more than 1000 mg/dL (720 mg/dL to 1430 mg/dL IgG level for maintaining an infection-free state) has been known as the optimal concentration needed to provide patients with protection against infections (29). Furthermore, subcutaneous immunoglobulin (SCIg) replacement can be considered as a suitable method for the stabilization of IgG levels, the improvement of health-related quality-of-life scores, and minimizing systemic side effects with increased ease of administration and affordability in comparison with intravenous immunoglobulin (IVIG) replacement (159–163). Moreover, antimicrobial prophylaxis including *levofloxacin* for gram-negative bacteria in patients with neutropenia can be beneficial in the prevention of bacterial infections in patients receiving CD19-redirected CAR-T therapy (25, 164, 165). Similarly, antifungal prophylaxis such as *micafungin* or *fluconazole* for Candida species in patients with neutropenia and *pentamidine* or *sulfamethoxazole* for *Pneumocystis jiroveci* are among common recommendations for fungal infection prevention (25, 164, 165). Also, in the case of viral infections, antiviral prophylaxis such as *acyclovir* is used for controlling herpes simplex virus (HSV)- and varicella-zoster virus (VZV)-related viral infections after CD19-redirected CAR-T therapy (25, 164, 165). Moreover, a recent study has reported prolonged severe acute respiratory syndrome coronavirus 2 (SARS-CoV-2) infection in a patient who had received BCMA-redirected CAR-T therapy (166). Hensley et al. stated that even though convalescent plasma therapy and the antiviral agent *remdesivir* was used as treatment options, the patient ultimately died from complications related to this infection (166).

### 3.4 Overcoming Unintentional Transduction of Leukemic Cells

**Figure 3:**
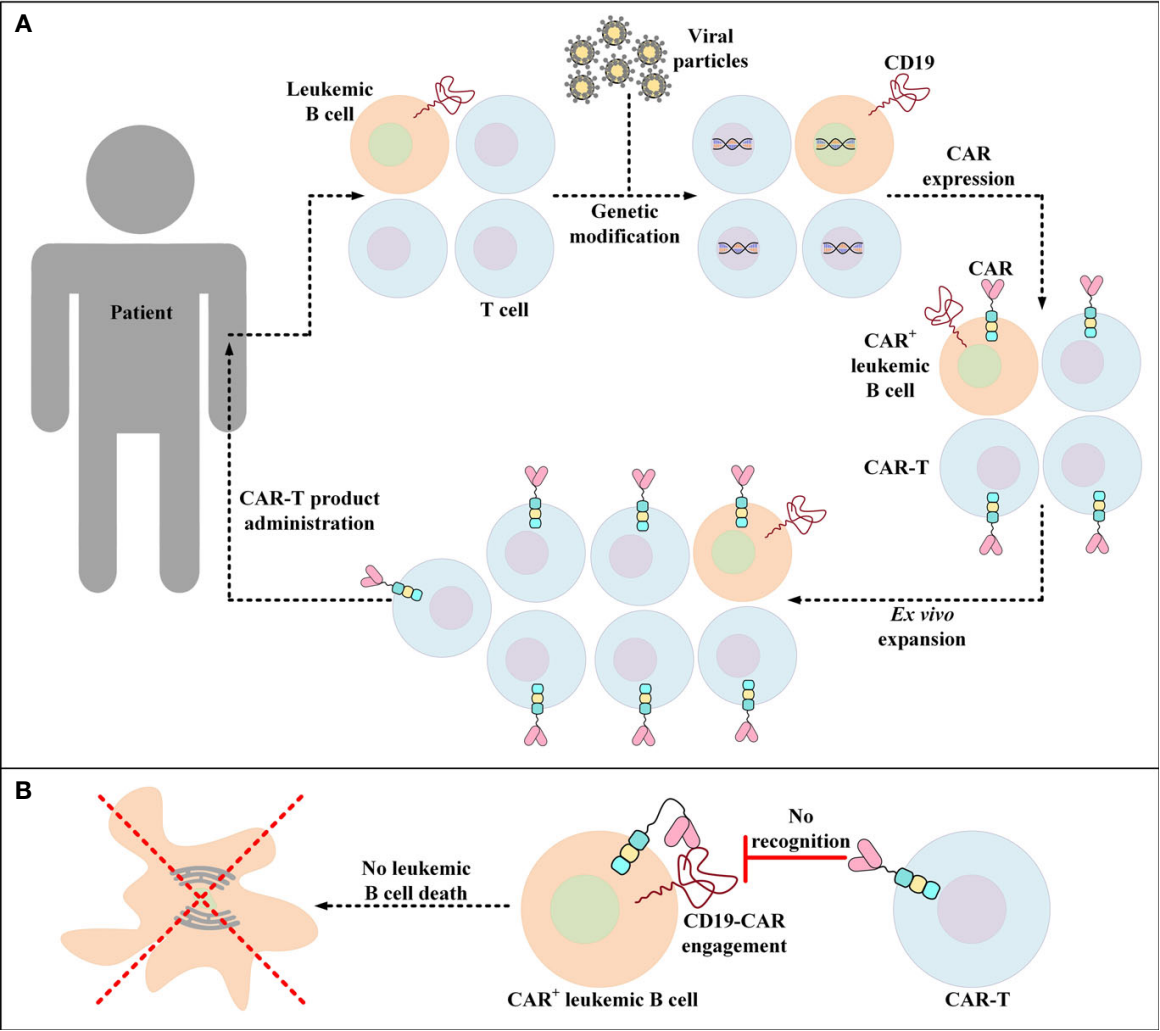
Accidental transduction of leukemic B cells and the emergence of resistance to CAR-T therapy. **(A)** Accidental transduction of leukemic B cells during the process of CAR-T preparation. An error in the isolation of T cells leads to the contamination of the cell pool with leukemic B cells. Moreover, in the process of genetic modification, leukemic B cells are transduced along with the isolated T cells for the expression of certain CD19-specific CARs. **(B)** Resistance of CAR^+^ leukemic cells to a particular CD19-redirected CAR-T product. Following the administration of the developed product into patients, CAR^+^ leukemic cells become resistant to that particular CD19-redirected CAR-T product due to the engagement of their CD19 with their self-expressed CARs (a process known as *epitope masking*). This incident might also occur in the case of CAR-T products that target different target antigens than CD19.

Unintentional transduction of B-ALL blasts with the CAR transgene during the manufacturing process of CAR-Ts is a rare incidence that leads to the aberrant expression of the CD19-redirected CARs by leukemic cells rendering them resistant to CAR-T-mediated tumor cell eradication (**Figure 3**) (167). Upon this incidence, a mechanism of resistance is conferred through cis binding of the CD19-redirected CAR to the CD19 epitope on the surface of leukemic cells, thus masking the target antigen from being recognized by CD19-redirected CAR-Ts (167). Therefore, the patient in which this incidence was documented for the very first time experienced CD19^–^ disease relapse and underwent salvage chemotherapy, anti-CD22 antibody therapy, and CD22-directed CAR-T therapy (167). However, the patient ultimately died from the complications associated with progressive leukemia (167).

Later in 2020, Ruella et al. developed an anti-CAR19 idiotype CAR (αCAR19) to selectively target and eliminate CD19-redirected CAR^+^ cells (both T cells and unintentionally transduced leukemic cells) (168). This method entails the application of genetically modified T cells (αCAR19) that express CARs that recognize CD19-redirected CAR^+^ cells (168, 169). It has been demonstrated that the αCAR19 T cells can successfully kill both CD19-redirected CAR-expressing leukemic cells and T cells (168). It is worth mentioning that no evidence of reverse killing of αCAR19 T cells by CD19-redirected CAR-expressing T cells has been observed (168). These findings demonstrated that αCAR19 T cells can act as “*cellular antidotes”* and a safe depletion strategy to eliminate common CAR-T engraftment side effects such as prolonged B-cell aplasia and other yet-to-be-known complications as well as CAR-expressing leukemic cells (168, 170). However, broader investigations are still required to safely conclude that this strategy may be efficient in controlling this important unintentional side effect.

### 3.5 Phenotype-Changing Factors

**Figure 4:**
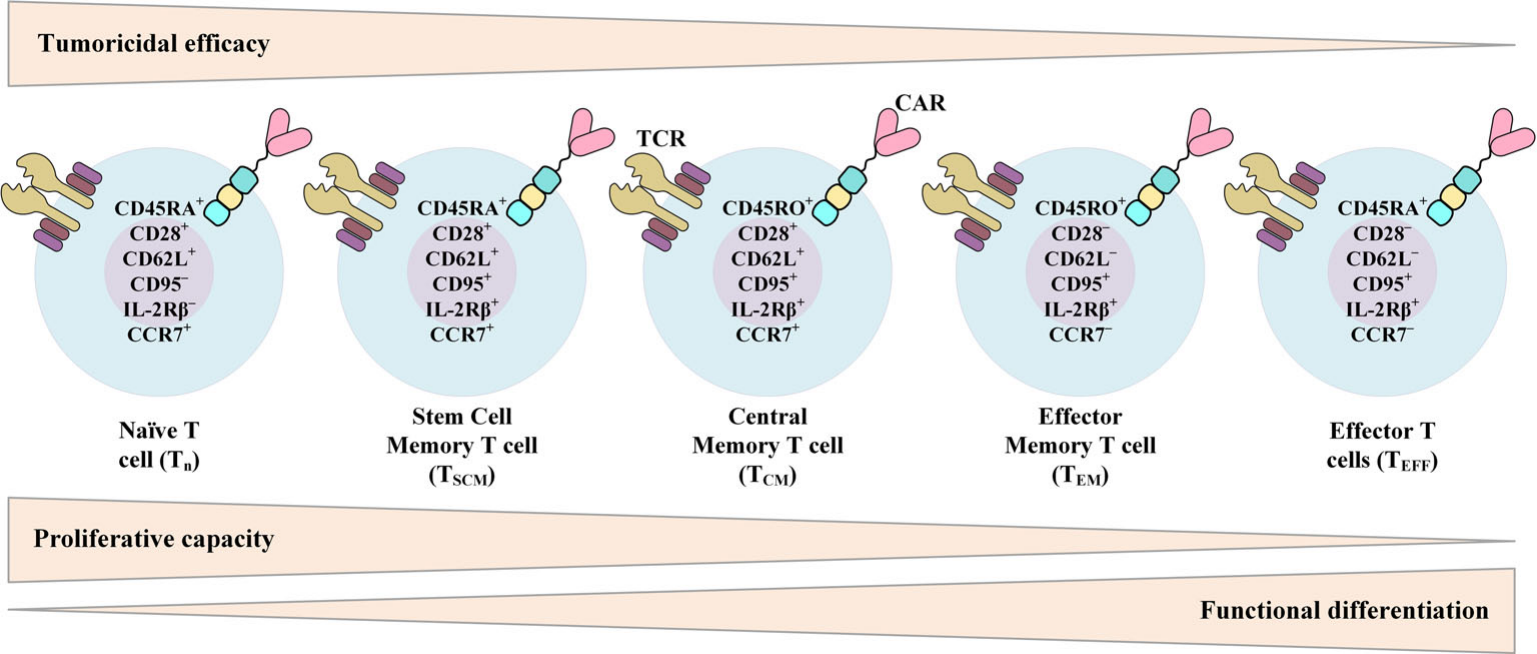
Phenotypic characteristics of different T-cell (or CAR-T) subsets over the course of T-cell differentiation from naïve T cells to effector T cells (T_EFF_) and their relationship with the proliferative capacity, functional differentiation, and tumoricidal efficacy of T cells. CAR, chimeric antigen receptor; TCR, T-cell receptor.

The data from various studies propose that there is a strong correlation between the expansion rate of transferred CAR-Ts in the recipient and the subsequent clinical response (14, 24–27). It is also believed that the *in vivo* persistence of functional CAR-Ts is critical for disease relapse prevention (14, 24–27). The phenotypic composition of patient- or donor-isolated T cells and the reinfused product is among multiple factors affecting the *in vivo* expansion and persistence of CAR-Ts and the durability of their antitumor responses (**Figure 4**) (171). In this section, we will discuss various factors affecting the phenotype of CAR-Ts.

#### 3.5.1 CD4^+^:CD8^+^ Ratio

Different investigations have demonstrated that human CD4^+^ T cells and CD8^+^ T cells consist of functionally and transcriptionally separate subsets with varied *in vivo* proliferation and persistence capacities after *ex vivo* expansion and adoptive transfer (172–177). Sommermeyer et al. have shown that human CD19-redirected CAR-Ts manufactured from purified CD8^+^ or CD4^+^ central memory T (T_CM_) cells or naïve T (T_N_) cells are more effective in attacking and killing CD19^+^ tumors in immunodeficient mice in comparison with CD19-redirected CAR-Ts manufactured from effector memory T (T_EM_) cells (178). Other studies have also demonstrated that in comparison to T_EM_ cells, CD8^+^ T_CM_ cells harbor stemness potency alongside showing superior persistence after adoptive transfer (172, 179). This fact proposes the advantages of this subset for providing long-term persistence (172, 179). Moreover, in the first clinical trial assessing the feasibility of selecting and engineering defined T-cell subsets, Turtle et al. conducted a Phase I/II clinical trial to evaluate CD19-redirected CAR-Ts manufactured from separately modified defined CD4^+^ T-cell and CD8^+^ T-cell subsets (180). These researchers administered this product in a defined CD4^+^:CD8^+^ composition and in a dose-escalation/de-escalation format to adult B-ALL patients after lymphodepletion chemotherapy (180). They found that the infusion of CAR-T comprised of a uniform ratio of CD4^+^:CD8^+^ shows a correlation between cell dose and earlier and higher peak expansion of clonally diverse CAR-Ts (180). These findings had not been reported in other investigations in which CAR-Ts were manufactured and infused without the consideration of the CD4^+^:CD8^+^ ratio (180). Their results also demonstrated that the infusion of a defined ratio of CD19-redirected CAR-Ts manufactured from CD8^+^ T_CM_ cells and CD4^+^ T cells could provide a synergistic improvement in potency (180). Also, the results of preclinical investigations in Raji tumor-bearing immunodeficient mice receiving CAR-Ts manufactured from distinct T-cell subsets have shown that when either CD8^+^ CAR-Ts or CD4^+^ CAR-Ts were excluded from the formulated product, the potency of the infused product was significantly reduced (178). It is safe to say that selecting a defined subset of T cells for CAR-T manufacturing with a uniform composition may provide reproducible potency in clinical therapy (180). Moreover, Biasco et al. have recently highlighted the impact of different clonal subtypes of T cells in the final CAR-T product on early antitumor responses and long-term disease-controlling capability of CAR-T therapy (181). These researchers stated that even though T memory stem cells (T_SCM_) have a low frequency in the final generated CAR-T product, these T-cell clones significantly contribute to the circulating CAR-T pools throughout both early proliferation and prolonged disease-controlling persistence (181). As suggested by Biasco et al., these findings underscore the significant role of T_SCM_ in both early anti-leukemic CAR-T reactions and prolonged disease control (181). However, it is important to mention that CAR-T prolonged persistence, after achieving the desired clinical outcomes and disease control, might lead to cytopenia which should be taken into consideration (182).

#### 3.5.2 *Ex Vivo* Culture Media

The different cell culture media formulations used for the activation and expansion of T cells *ex vivo*, and eventually generating CAR-Ts can be optimized in many ways since they tend to have significant impacts on various aspects of the final T-cell product (183, 184). The cell culture media formulations currently used are dependent on fetal bovine serum (FBS) or human serum (HS) (183, 184). Researchers have investigated the effects of the human transfusion-grade whole blood fraction-derived concentrated growth factor extracts, named Physiologix™ xeno-free (XF) hGFC (Phx), on CAR-T expansion and function (183, 184). This method has been developed to resolve the supply issues of FBS or HS (183, 184). It has been found that Phx enhances T-cell proliferation in both research and clinical-grade media and improves lentiviral-mediated gene transferring and expression (183, 184). Moreover, CAR-Ts expanded *ex vivo* in Phx-conditioned media harbor advanced *in vivo* expansion and antitumor activity as compared to those of CAR-Ts expanded *ex vivo* in HS-conditioned media (183, 184). Phx also enhances the number of naïve T cells (CD45RO^-^/CCR7^+^) and central memory T cells (CD45RO^+^/CCR7^+^) in both CD4^+^ T-cell and CD8^+^ T-cell populations which contributes to higher persistence and durability of CAR-Ts resulting in better clinical outcomes (183, 184). Geiger and colleagues have also found that, upon T-cell activation, significant changes occur in the arginine metabolism resulting in a decline in the concentration of the intracellular L-arginine (185). Therefore, these researchers demonstrated that supplementing T-cell culture media with L-arginine can increase the reduced intracellular L-arginine levels in activated T cells and it can induce oxidative phosphorylation instead of glycolysis (185). This effect can result in the central memory phenotype development (185).

The methods currently used for generating FDA-approved CAR-Ts include using anti-CD3 and IL-2 or anti-CD3 and anti-CD28 beads (186). However, these methods result in the generation of CAR-Ts expressing exhaustion markers with an effector phenotype (186). Studies have demonstrated that using IL-7 and IL-15 during the *ex vivo* culturing of T cells would result in the production of CAR-Ts with T memory stem cell phenotype (CD45RA^+^/CCR7^+^) that exhibit superior tumoricidal activity, expansion, and persistence *in vivo* as compared with their counterparts expanded *ex vivo* using IL-2 (187). Others studies have also reported very similar results alongside adding that using IL-7 and IL-15 during the *ex vivo* culturing of T cells also elevates the response rate of the resultant CAR-Ts to anti-PD-1 adjuvant therapies which is mainly due to the anti-PD-1-responsive CD8^+^/CD62L^+^/TCF7^+^/IRF4^–^ population of the CAR-Ts (188). However, Alizadeh et al. have indicated that T cells expanded with IL-15 preconditioning maintain a less differentiated stem cell memory phenotype (CD62L^+^/CD45RA^+^/CCR7^+^) with reduced levels of exhaustion marker expression in comparison with T cells preconditioned with IL-2. They have also stated that using IL-7 or IL-21 alongside IL-15 decreases the favorable influences of IL-15 in the induction of CAR-T phenotype and tumoricidal activity (189).

Additionally, it has been demonstrated that using human platelet lysate (HPL) as a supplement in the culture media of T cells results in a higher number of T cells with central memory phenotype in comparison with human AB serum (ABS)- or FBS-supplemented culturing media (190). It has also been discovered that the presence of various cytokines, such as IL-7, in HPL is responsible for the phenotypic differences observed in the mentioned study (190). Furthermore, Torres Chavez et al. have also obtained similar results indicating that HPL-supplemented *ex vivo* culturing media support T cells to preserve their less differentiated cell phenotype resulting in their longstanding antitumor activity and persistence (191). Above all this, some researchers have demonstrated that treatment of mouse CD8^+^ T cells with the S enantiomer of the 2-hydroxyglutarate (S-2HG) in *ex vivo* culture significantly enhances their *in vivo* expansion, persistence, and tumoricidal activity (192). Recently, the same group has indicated that S-2HG acts as an immune metabolite and helps clinical-grade allogeneic CAR-Ts maintain their central memory phenotype and exhibit enhanced antitumor activity (193).

#### 3.5.3 *Ex Vivo* Culture Duration

According to the relationship between the state of T-cell differentiation and the potential for *in vivo* engraftment and persistence, some researchers have proposed that reducing the duration of *ex vivo* culturing can limit differentiation and significant loss of stemness and enhance the efficacy of CAR-T therapy (194). Ghassemi et al. have demonstrated that reduced culturing duration helps CAR-Ts have improved *ex vivo* expansion and effector function which are directly correlated with enhanced *in vivo* engraftment and tumoricidal activity (194). These researchers also demonstrated that these CAR-Ts eradicated human B-ALL in a murine xenograft model even at a 6-fold lower dose as compared with their counterparts expanded with the conventional *ex vivo* durations (194). In a nutshell, Ghassemi et al. demonstrated that the antileukemic activity of CAR-Ts is inversely correlated with their *ex vivo* culturing duration (194).

#### 3.5.4 Other Factors

Upon activation, T cells steer their metabolism from fatty acid oxidation to glycolysis which helps them keep their effector function (195). It has been found that T cells with a memory-like phenotype have a low level of glucose uptake in comparison with T cells with an effector-like phenotype (195). Sukumar et al. have demonstrated that activating CD8^+^ T cells in the presence of 2-deoxyglucose, which is a glycolysis pathway suppressor, can result in the formation of memory T cells (195). Moreover, other researchers have shown that pharmacologic inhibition of the serine/threonine kinase Akt promotes the expansion of TILs with transcriptional, metabolic, and functional features of memory T cells (196). Furthermore, Urak et al. also highlighted the Akt pathway involvement in T-cell differentiation state and memory phenotype formation (197). They demonstrated that the inhibition of Akt using an Akt inhibitor during *ex vivo* expansion of CAR-Ts leads to the production of CAR-Ts expressing higher levels of CD62L and CD28 as compared with CAR-Ts generated without Akt inhibition (197). Moreover, these researchers reported that *ex vivo* Akt-inhibited CAR-Ts exhibit superior antitumor functionality both *in vivo* and *in vitro* as compared with conventional CAR-Ts (197).

Lately, it has been found that the differentiation fate of CD8^+^ T cells is regulated by the methylcytosine dioxygenase ten-eleven translocation 2 (TET2) (198, 199). TET2 loss results in the formation of memory-like phenotype in CD8^+^ T cells without any impairment of their expansion or effector function (198, 199). Moreover, TET2-disrupted CAR-Ts have exhibited central memory phenotype at the expansion peak, which highlights the beneficial impact of TET2 suppression in CAR-Ts (198, 199). Furthermore, Kondo et al. have demonstrated that activated effector T cells can be converted into T_SCM_-like (iT_SCM_) cells by co-culturing with OP9 stromal cells expressing a Notch ligand such as Delta-like 1 (200–202). It has been found that using IL-7 and IL-15 can significantly be beneficial for the production of iT_SCM_ cells (201). These researchers also indicated that iT_SCM_ cells are tolerance-resistant alongside exhibiting robust engraftment, persistence, and tumoricidal activity *in vivo* (202). More recently, Kondo et al. reported that conventional human CAR-Ts can also be converted into iT_SCM_ CAR-Ts *via* Notch signaling-mediated mitochondrial metabolic reprogramming (203). Additionally, they found that Forkhead box M1 (FOXM1) is involved in the downstream signaling of the Notch pathway (203). FOXM1 is mainly responsible for the metabolic reprogramming of T cells and stem cell memory phenotype induction, and iT_SCM_ CAR-Ts generated using FOXM1 induction are as functional as iT_SCM_ CAR-Ts generated using Notch induction (203). In addition to the mentioned strategies, it is known that the 4-1BB signaling promotes T-cell central memory phenotype formation and preservation in CAR-Ts by promoting oxidative metabolism (204). Therefore, using the 4-1BB co-stimulatory domain in the construct of CAR-Ts helps preserve their central memory phenotype and *in vivo* expansion capacity (46).

## 4 Conclusion

CAR-T therapy will just be another unfulfilled promise without optimized efficacy and efficient toxicity management strategies. This type of therapy offers a new fighting tool with improved targeting capacity capable of fighting R/R B-ALL with durable outcomes (12–20). As outlined in this review, the unfavorable adverse events of B-ALL CAR-T therapy appear as the leading obstacle in the way of its broader therapeutic benefit; therefore, an ideal balance between safety and efficacy is critically required (59). Rapidly evolving research advances in genomic editing methods alongside a better understanding of the mechanisms underlying the discussed toxicities will help us further prevent or manage CRS and neurotoxicity. Moving forward, having a combination of different strategies or an all-in-one unified approach for producing off-the-shelf allogeneic CAR-Ts can decrease the duration of CAR-T production and delivery and eliminate other limitations regarding the generation of autologous CAR-Ts using T cells isolated from heavily treated R/R B-ALL patients (205). A magnificent deal of effort has now been dedicated to these strategies since they can be beneficial for CAR-T therapy in both hematologic malignancies and solid tumors. However, some of these strategies are still in their infancy, as others have been evaluated in preclinical and clinical studies. Furthermore, other aspects of CAR-T therapy can also be improved for having a better CAR-T persistence and antitumor activity by controlling the factors that affect the phenotype of the final T-cell product. Despite all this, the financial limitations and disease relapse mechanisms can still be named as other limitations of B-ALL CAR-T therapy requiring counterstrategies.

## Author Contributions

PouS: Conceptualization, Investigation, Writing - original draft, Writing - review & editing, Validation, Supervision. PooS: Conceptualization, Investigation, Writing - original draft, Writing - review & editing, Validation, Supervision. FR: Writing - review & editing, Validation. All authors contributed to the article and approved the submitted version.

## Conflict of Interest

The authors declare that the research was conducted in the absence of any commercial or financial relationships that could be construed as a potential conflict of interest.

## Publisher’s Note

All claims expressed in this article are solely those of the authors and do not necessarily represent those of their affiliated organizations, or those of the publisher, the editors and the reviewers. Any product that may be evaluated in this article, or claim that may be made by its manufacturer, is not guaranteed or endorsed by the publisher.
